# Seeking Balance in Motion: The Role of Spontaneous Free Play in Promoting Social and Emotional Health in Early Childhood Care and Education

**DOI:** 10.3390/children1030280

**Published:** 2014-10-01

**Authors:** Jane Hewes

**Affiliations:** Early Learning and Child Care, Faculty of Health and Community Studies, MacEwan University, Edmonton AB T5J4P2, Canada; E-Mail: hewesj@macewan.ca; Tel.: +1-780-497-5193; Fax: +1-780-497-5848

**Keywords:** spontaneous free play, playfulness, social and emotional health, early childhood care and education, rough and tumble play, dizzy play

## Abstract

There is accumulating scientific evidence of the potential of play and playfulness to enhance human capacity to respond to adversity and cope with the stresses of everyday life. In play we build a repertoire of adaptive, flexible responses to unexpected events, in an environment separated from the real consequences of those events. Playfulness helps us maintain social and emotional equilibrium in times of rapid change and stress. Through play, we experience flow—A feeling of being taken to another place, out of time, where we have controlled of the world. This paper argues that spontaneous free play, controlled and directed by children and understood from the child’s perspective, contributes to children’s subjective experience of well-being, building a foundation for life-long social and emotional health. The paradoxical nature of young children’s spontaneous free play is explored. Adaptability, control, flexibility, resilience and balance result from the experience of uncertainty, unpredictability, novelty and non-productivity. These essential dimensions of young children’s spontaneous free play typically produce play which is experienced by adults as chaotic, nonsensical and disruptive. The article concludes with a preliminary discussion of the challenges and possibilities of providing for spontaneous free play indoors, in early childhood care and education programs.

## 1. Introduction

Any discussion of young children’s health would be incomplete without some reference to the role of play. Early childhood development is a determinant of health [1]. There is compelling neuroscientific evidence of the significance of early experience to life-long mental, as well as physical health [2], and accumulating evidence of the adaptive value of play and playfulness in the development of social competence, emotional resilience, and flexibility in response to unpredictability and stress [3,4]. The child is a citizen with the right to play [5]. There is an urgent need to articulate the conditions that ensure the young child’s right to spontaneous free play in the increasingly formal environments in which early childhood experience unfolds.

Profound changes in the physical and social environments of early experience in the western world are constraining opportunities for spontaneous free play in early childhood [6]. Changes in the demographics of family life are accompanied by an institutionalization of free play: young children are spending long hours in non-parental group care [7]; the majority of families now lives in cities, where traffic and urban land-use patterns have changed the natural play territory of childhood and children are less likely to have access to outdoor play spaces in natural environments; families are increasingly concerned about neighborhood safety, and are choosing to enroll young preschool children in structured education, recreation, and organized sports programs, leaving little time for self-initiated play [6]. There is growing concern amongst academics, professionals, policy makers and community leaders alike that the decline in free play opportunities may be a contributing factor to increasing rates of childhood obesity, and to the alarming increase in the incidence of anxiety, stress and depression in young children [8,9,10,11,12,13,14].

Neuroscientific evidence of the significance of early experience not just to individual health, but also to the long term social and economic prosperity of society as a whole [1,15] is driving a new public policy agenda in early childhood development. The evidence highlights the interconnectedness of physical, intellectual, social and emotional development [16], and of physical and mental health. There is powerful evidence about the impact of excessive stress and adversity in the early years on the incidence of a range of chronic diseases in adulthood [17], creating a new emphasis on the importance of social and emotional health in early childhood [2] and growing public policy interest in early intervention with children living in families coping with the stresses of poverty, violence, mental illness, and substance abuse. Early childhood is on the public policy agenda, and the environments where children spend time in their pre-school years are under intense scrutiny [18].

The focus of the public policy agenda in Canada and much of the Western world is on combatting sedentary lifestyles and childhood obesity, increasing self-regulation and impulse control, and ensuring school readiness, particularly for young children whose development may be compromised by social and environmental factors. This agenda is instrumentalizing [19,20] and pedagogizing [21] children’s play in the service of adult goals. Taking a playful approach to learning can be very effective, however it frequently takes control of the play away from the players, changing the experience for the player. However well-intentioned, the intensity of the adult agenda for early childhood, in combination with the changing social reality of early childhood experience, is crowding out children’s opportunities for spontaneous free play, an approach that undermines the benefits of play, and ultimately limits its potential to contribute to health [18]. Even very young children are now losing the opportunity to play for their own purposes and with this, essential opportunities to build the foundation of play, and playfulness, which determine in large part their subjective sense of well-being and belonging in childhood and may well affect their physical and mental health long into the future. 

This paper explores the potential of spontaneous free play controlled and directed by children themselves, to contribute to children’s experience of a healthy childhood and subjective well-being in the present, as well as to the foundation of life-long social and emotional health. The paper argues that opportunities for spontaneous free play, in particular spontaneous free play that allows young children to explore those dimensions of play which may appear to be chaotic, nonsensical and disruptive to adults, is key to their health and well-being. The barriers, challenges and possibilities of providing for these kinds of spontaneous free play opportunities in group early childhood care and education settings are explored.

## 2. Spontaneous Free Play and Health

Article 31 of the United Nations Convention on the Rights of the Child (UNCRC) challenges us to understand play from the perspective of the child, as a positive factor in children’s lives, and from their perspective, as a “necessity of life” [22] (p. 469). This section of the paper discusses the foundation for viewing young children’s spontaneous free play as necessary to their subjective sense of well-being in childhood, and considers the evidence that it also contributes to a foundation for social and emotional health and resilience over the life-span.

Section 2.1 considers both recent and established theoretical and conceptual writing defining the nature and characteristics of spontaneous free play, in order to distinguish it from other play-based approaches. Understanding play from the perspective of the player and children’s purposes in play is critical to understanding its benefits to social and emotional health. Section 2.2 looks more specifically at the disruptive dimensions of play in relationship to the emerging evidence about the role of spontaneous free play in developing flexibility and adaptability, building the capacity to cope with everyday stress and anxiety. The body of knowledge coalescing around the value of rough and tumble play in healthy social and emotional development in early childhood provides a focus for understanding how children play with the ongoing social and emotional self-balancing that is fundamental to successful participation in social life.

### 2.1. The Nature of Play and Spontaneous Free Play in Early Childhood

“Play is a thing by itself” [23] (p. 45). The recognition of play as a distinct conceptual category and an irreducible concept in human culture rings true nearly 75 years after the publication of *Homo Ludens: A Study of the Play Element in Culture.* Huizinga emphasizes that play cannot be reduced to other terms or understood by connecting it to a functional purpose that is not play, arguing that to be understood, play must be seen as the player(s) sees it [23] (p. 21). Seeking always to understand the perspective of the player—the child’s purposes in playing—is critical to understanding the social and emotional benefits of spontaneous free play in early childhood. For purposes of this paper, it is important to consider some of the defining characteristics of spontaneous free play in childhood, in order to distinguish it clearly from notions of “educational play”, “guided play”, and “purposeful play”, which have crept into recent academic and policy literature, and to highlight some of the key features of spontaneous free play that speak to its value in promoting social and emotional health in early childhood.

The defining characteristic of spontaneous free play is the control of the play by the players. This is the source of some of its unique benefits for children, and most enduring challenges for adults. Children, particularly young children, are never really in control of their everyday lives. Nonetheless, the experience of being in control and making decisions in play can contribute to children’s understanding of themselves as social actors and active participants in determining the course of their daily lives. Although play is not the only experience that affords children an opportunity to make their own decisions, in spontaneous free play controlled by the players, children can explore what it means to be in control of themselves and others, without the full responsibility of being in control. Importantly, play also offers a context where children can explore being out of control, in ways that are often unacceptable outside of the play context. For the child, play and playing is fundamentally about agency, power, and control. In play, children actively explore their own social and physical power, in relationship to the world, and to other children. As each child participates with other children in the social contexts of play, exploring and testing and making decisions at the edges of their own possibility, they come to understand what it means to be in control, and what it means to be out of control. When left to control their own play, they do explore what it means to exert their own power over others, and they do take chances and physical risks. These are essential dimensions of spontaneous free play that present critical ethical challenges for adults. It is worthy of note that the notions of participation and control are deeply embedded in the language of health promotion [24]. Active participation in community and in particular in the decisions that affect us contributes to a sense of control over the multiple factors that influence not just our physical and mental health, but also our subjective sense of well-being and belonging.

The question “What is play?” both plagues and fascinates researchers, writers, philosophers, poets, parents and educators. Children, arguably the experts on play and playing, seem to know exactly what it is and are unconcerned with trying to call it anything else. As play theorist Brian Sutton-Smith observes, “We all play occasionally, and we all know what playing feels like. However, when it comes to making theoretical statements about what play is, we descend into silliness” [25] (p.1). 

Play has a pervasive and ubiquitous presence in human culture and across multiple animal species. We tend to associate play and playfulness with the young of a species. While the play of children, kittens, puppies, and monkeys is familiar, animal play researchers have also observed playful behavior in birds, turtles, fish and even some insects [26]. Despite its variability and remarkable social complexity in the animal world, play seems to be more clearly identifiable as a distinct behavior in animals than it does in humans. The message “this is play” [27] as well as the invitation to play, is behaviourally clear in many animal species. When a puppy wants to play, it assumes a characteristic pose, lowering on its front legs and wagging its tail. The invitation to play in young children is often much more ambiguous, requiring subtle interpretation of social communication, as well as a certain kind of emotional resiliency, as the following example of two boys meeting on a public playground for the first time, illustrates:

Boy #1 (opening the conversation and for no apparent reason): You’re *a baby*
Boy #2: No, *you’re* a baby
Boy #1: You’re a *ridiculous* baby
Boy #2: *You’re a poop stick!*
Boy #1: Do ya wanna play [28] ?

Human play is arguably more varied and complex than animal play. There are many forms of childhood play and an equally diverse array of functions of human play as are emerging in the study of animal play. It is this sheer diversity of types and functions of play that make it difficult to define. Burghardt notes that the diversity “obscure[s] the commonalities of all forms of both human and animal play” [29] (p. 341). Play defies definition and eludes categorization at every turn; the more we try to pin it down, the more it moves—play is playful—it is a “hobgoblin” [23].

Traditional play classification schemes fall apart in the face of a sustained episode of young children’s spontaneous free play, which is by nature combinatorial [30], and may exhibit multiple forms, types and stages simultaneously. A recent observation of a group of five year old boys deeply engaged together in block play confirms that children continually slip in and out of solitary, onlooker, parallel, associative and cooperative play [31] as it suits their purposes, developing their own ideas and seeking out the possibilities and fun that results when their playfulness intersects with the play narratives and constructions of other players. Their play embraces aspects of object play, sensory-motor play, construction play, symbolic play, and games with invented rules [32]. The disruptive dimensions of play that are the subject of this paper—the rowdy, rambunctious, nonsensical, irrational, elements of rough and tumble, and order and disorder, both physical and verbal—are characteristic of spontaneous free play. These are better understood as dimensions and qualities of the spontaneous free play experience rather than forms of play, dimensions that coexist simultaneously and fluidly, forming, reforming, appearing and disappearing spontaneously, as any free play episode unfolds.

In early childhood education, children’s play is often described using some variation of the characteristics of play identified by Rubin, Fein and Vandenberg in 1983 [33], based on a review of psychological research on play. Play is variously described as voluntary, freely chosen and intrinsically motivated; controlled and directed by the players; possessing a non-literal, “as if” quality; being free of externally imposed rules, taking place ‘to the side of’ or ‘outside of’ the rules of ordinary life; undertaken for no immediate goal or purpose, and focused on means rather than ends; characterized by active engagement, deeply absorbing and satisfying for the players; and, generally speaking, producing a positive, pleasurable affect for the player. While this definition of play is in many ways explanatory, it does not capture essential elements and nuances of spontaneous free play from the perspective of the player. For example, the idea that play is free of externally imposed rules suggests, quite rightly, that children are able to make their own rules in play. What it does not adequately describe or explain is the meaning of play and playing that pushes the edges, challenging and even breaking the rules of ordinary life. The popular YouTube video [34] of a wild polar bear returning night after night to play with a husky sled dog is a stunning example of play that breaks the rules of ordinary life. Young children routinely challenge the rules in play, for example climbing up the slide rather than sliding down it is a playful approach which breaks the rules of how young children are expected to use slides in most early childhood care and education programs. The notion that children play to experience pleasure is also limited; when children play they feel powerful. Jumping from a playground platform is a total body encounter with gravity and an experience of the power of flying.

Stuart Brown, a psychiatrist and play advocate, adds depth to our understanding of the nature of spontaneous free play from the player’s perspective. He lists the following as properties of play: “apparently purposeless, voluntary, inherent attraction, freedom from time, diminished consciousness of self, improvisational potential, [and] continuation of desire” [35] (p. 17). The notion of a diminished sense of self is echoed in Csikzentmihalyi’s discussion of play as the “flow experience par excellence”, a “the merging of action and awareness” [36] (p. 37–38). A similar notion appears in Gadamer’s philosophical treatise on play: “Play fulfills its purpose only if the player loses himself in play” [37] (p.102). Along with the idea of being free from time in play, this notion may speak to the role of play in reducing stress, by taking the player outside of him/herself, into another reality, even for brief periods of time. The improvisational nature of play is thoroughly explored by Keith Sawyer in his study of preschool pretend play [38], a quality of play may be linked to its capacity to enhance adaptability and flexibility in response to rapid change.

Brown’s notion of the players’ “apparent” purposelessness in play and their desire to continue the play is essential in understanding young children’s purposes in spontaneous free play, particularly as it challenges the commonly held notion that spontaneous free play is goalless or purposeless. The players do have one key purpose, and that is to keep the play going, particularly if it is accompanied by pleasurable affect and feelings of power. Keeping the play going is the source of incredible creativity and spontaneous innovation in play. We observe young children introducing surprising novelty into story lines and character roles, in order to sustain the play or include more players or combine their play narrative with another group. Sutton-Smith proposes that these “quirky twists” are characteristic of play and may be connected to its potential to contribute to “adaptive variability” [25] (p. 229). Spontaneous free play can only take place in an environment where spontaneity is possible. It must be possible for play to “erupt” and take off in unusual directions, for metal pots to be hats in one moment and drums in a marching band in the next. It is common in young children’s play that these spontaneous narrative directions are non-linear, irrational and difficult for adults to follow. The phenomenon of group glee [39] in toddlers and preschoolers is another spontaneously disruptive and common feature of free play, one which produces a strong sense of social bonding and belonging. The shared humor of very young children is not obvious to adults and other outsiders. For the player, these experiences nourish feelings of subjective well-being in the here and now, and are now acknowledged as some of the immediate social and emotional benefits of children’s spontaneous free play [4] (p. 114).

In an attempt to distinguish play from nonplay behaviour in animals, Burghardt [29] (pp. 345–346) identifies several dimensions of play that shed further light on understanding spontaneous free play in early childhood. He describes the voluntary nature of play as being intentional, which further complicates our understanding of play as purposeless. Burghardt notes that the purposelessness of play in the animal world is specific to immediate survival needs. The purposelessness that adults observe in children’s play may not be shared by the child(ren). The players may be pursuing purposes that are neither immediately obvious nor purposeful from an adult perspective. Burghardt goes on to describe the exaggerated, novel, repetitive and incomplete behavioral patterns characteristic of animal play, which are reminiscent of young children’s playful ways of moving. While an adult will walk efficiently, a small child walking from the house to the car will adopt a gait that includes elements of hopping, skipping, and galloping. This expressive and playful approach to movement has been aptly described as “galumphing” [30], and is characterized by exaggeration, reordering and repetition of sequences of behaviour. Spontaneous free play frequently involves intentional, systematic and novel complication of behavioural patterns, building a combinatorial freedom and flexibility in the behavioural repertoire, arguably rendering both animals and humans more adaptable [40].

The following description of free play introduces the notion that some of the more disruptive qualities of play may be defining features of the spontaneous free play experience from the players’ perspective, whose main purpose in play quickly becomes to keep playing.

In spite of the complexity and diversity of play behaviour, there is general agreement by specialists in the field that play is controlled by children rather than by adults, and that it is undertaken for its own sake and not for prescribed purposes. The term “free play” is often used to distinguish this from organized recreational and learning activities, which also have important roles in child development. However, the characteristics of free play—control, uncertainty, flexibility, novelty, and non-productivity—are what produce a high degree of pleasure and, simultaneously, the incentive to continue to play [41] (p. 25). These qualities of free play produce the affect that the player is seeking, and which have the potential to contribute positively to children’s health and sense of well-being. They are also the qualities that lead to play that is frequently suppressed by adults because it tends to be disruptive [4] (p. 17). Interestingly, these are also the qualities of animal play that are understood to contribute to its adaptive value [42].

Children value play. It is significant in their lives. The awareness that children may have purposes in their spontaneous free play that are neither readily apparent, nor important to adults, is key to understanding the potential of play to contribute to the subjective experience of well-being in childhood. A critical difference between spontaneous free play and other play based approaches lies in the participation of the adult. Children make a very clear distinction about what is and is not play based on how adults participate [43]. In spontaneous free play, the locus of control remains with the players. Adult efforts to guide and direct play—either out of necessity or in the service of a developmental or learning agenda—generally interrupt the flow of the play for the player(s). The idea that the benefits of play accrue most directly from play where the frame is both set and sustained by the players themselves presents significant challenges to adult sensibilities and to the expectations of early childhood educators. For children, play must be spontaneous free play in order to be experienced as play. This means it is controlled and directed by children, even when adults are playing. Other kinds of play based approaches are neither experienced as play by children, nor defined by them this way. 

### 2.2. Promoting Social and Emotional Health: Making a Case for Disruptive, Disorderly, Dizzy Play

A theoretical explanation of what exactly is important about play, how its associated benefits are realized and what dimensions of play produce which benefits is almost as difficult as defining it. There is an extraordinary array of developmental and learning benefits that are associated with young children’s play, but the evidence linking these benefits to play remains largely correlational [44]. The benefits of animal play are most often linked with the rehearsal of behaviours that serve no immediate survival purpose, but, in the words of Karl Groos “will later be essential to life” [45]. Recent evidence from neuroscience suggests that play may have more important social, emotional, and affective benefits in the immediate context of living than were previously understood [4] (p. 15). As Pellis *et al*. note, [46] (p. 279), the multifunctional nature of play in animals and humans contributes to the difficulty in articulating a coherent explanatory theory as to its adaptive value. Nonetheless, researchers and play theorists alike maintain that there must be some adaptive value to play, given its pervasiveness, its ubiquity and its evolutionary resilience. 

This section of the paper looks specifically at the potential of play to contribute to social and emotional health in early childhood, building a case for the idea that the power of play to make us resilient, flexible, and strong—emotionally, socially, physically, intellectually, and perhaps spiritually—may lie in its propensity to invert and subvert the order of things. In particular, it is argued that those dimensions of young children’s spontaneous free play and playfulness that experiment with ordering, disordering, and reordering, rough and tumble, and all forms of dizzy, chaotic, nonsense play may provide critical opportunities for children to experience a sense of social belonging, well-being and participation in the culture of childhood, as well as to develop social and emotional awareness, control and resilience. Play helps children learn to “roll with the punches” of everyday life [42,47], and to experience the ongoing social and emotional balancing of self that is fundamental to successful participation in social life. 

The similarities between health promotion action strategies—strengthening social relationships, personal control and participation [24] and the characteristics of spontaneous free play, support the notion that play can be understood as a contributing factor to health in its broadest sense. Participation in the peer culture of play is foundational to the child’s sense of well-being and belonging in childhood; play creates the possibility of control, motivating and challenging the player to explore the dimensions of their own agency, and thereby experiencing the emotional reality, the thrill, and the risk of making decisions, in an environment that is buffered from the real consequences of those decisions. Play is behavior in the “simulative mode” [48]. In a recent analysis of the decline of free play in childhood, Peter Gray reviews the evidence linking the loss of a sense of personal control and the lack of social connectedness to the increasing incidence of depression and anxiety in children and young adults [12] (p. 449). These are critical dimensions of health promotion action strategies and also key features of spontaneous free play. Play promotes health in early childhood. 

The impact of vigorously active spontaneous free play on young children’s physical health—on their strength, coordination, spatial awareness and balance—is readily observable. Less obvious, but now quite compelling, is the thought that this same kind of playful exploration of balance and balancing that characterizes so much of young children’s physical play, might also be going on in relationship to the emotional realities and social relationships that are so much a part of the play material of childhood, and that there might be similar benefits to emotional resilience, social awareness, the coordination of self with others [49], and the ability to maintain social and emotional equilibrium when things are changing. 

#### 2.2.1. Spontaneous Free Play Alleviates Stress

Young children use play to cope with stress in situations of extreme trauma as well as everyday events. There is considerable concern with the impact of stress and adversity in early childhood on the developing brain, primarily with the profound impact that extreme adversity or toxic stress in early childhood (resulting from violence, neglect, or abuse), has on life-long physical as well as mental health [17]. The evidence is suggesting that the developing brain needs just the right amount of the right kind of stress; there is concern about the impact of removing all stress from children’s lives, as well as the supports that should be in place for young children to cope with every day or tolerable stress, for example, with the stress of moving to a new home, or beginning school. There is some common sense in the notion that playing, similar to other forms of recreation, alleviates stress. 

The player’s experience of play is often associated with freedom. Play is freeing; it frees us temporarily from the cares and stresses of everyday life. As noted previously, and importantly, play is a place where the player feels in control of the world, even for a brief time. It is a hopeful place. Play is about possibility, and for a time, anything can be possible in play. Sutton Smith asserts that the opposite of play is depression [25] (p. 198). Gadamer argues that play is experienced by the player as being effortless and without strain [37] (p.105); the result, he claims, is relaxation. The common sense is that play helps the player to relax, which can be beneficial in coping with stress. The notion of playfulness is important to consider, in addition to play. As illustrated in the fable of the oak and willow, the flexibility that characterizes playfulness can also be adaptive. 

However, the relationship between play and stress and how each impacts the other appears to be much more complex than common sense would have it, and like other aspects of play, is informed by multiple disciplinary perspectives. Animal neuroscience [50] is enhancing our understanding of the relationships between stress, adversity, brain development and play. Play is impacted by stress, *i.e*., situations of anxiety and threat reduce play and playfulness, but play also appears to be resilient to stress, with some evidence that some stress may facilitate play [29]. The research on play in psychotherapy reveals its considerable power as a cathartic and expressive outlet for young children who are coping with overwhelming, confusing, traumatic emotions and life events. In play therapy, children replay and/or play out fearful or stressful situations, often repeatedly, in order to gain control over the emotions. Play has been used effectively to help children cope with hospital stays [51] and surgery, as well as to cope with the aftermath of natural disasters [52]. 

Young children frequently use play to work through everyday stresses and anxieties. Finding out that daddy is going away for a few days, that grandma is picking you up today instead of Mom, or that your brother will not be coming to child care today because he is sick, are common examples of the everyday stresses that young children gain emotional control of through play. Interestingly, there is some research that suggests that children use solitary play to work through these emotions more often than social play, and that this kind of play can be quite repetitive [53]. Noticing the child’s reality and protecting the time and space needed for this kind of play in a busy early childhood program can be challenging. There is a tendency to intervene in play that is repetitive, without first asking what the purpose of the repetition might be. The repetitiveness of this kind of play is arguably linked to establishing a sense of predictability and control. A recent play scenario of a toddler whose father was going away for a few days was captured on video and serves as an interesting example of the everyday potential of play to help children cope with stress and work through normal anxieties. Shortly after her father dropped her off at child care, the child sat down to play at a dollhouse. She played by herself, quite contentedly, inventing conversations between the family figures for well over 15 minutes, which many might consider an unusual length of time for a child of this age. What is audible on the video is her repetition of the phrase “You ok? You ok?” A follow-up conversation with the child care educator confirms that this was part of the conversation she had with her father at drop off. The child is actively seeking to balance her emotional reality through the expressive medium of play. After Geertz, she is using play to tell herself a story about herself [54] (p. 674). 

#### 2.2.2. The Social and Emotional Value of Disruptive, Disorderly, Dizzy Play

Play works in fundamentally paradoxical ways, and it is not always what it seems. Young children have a preponderance for dizzy play, most obvious in their persistent pursuit of vertigo—spinning, whirling, swiveling, twirling, somersaulting and tumbling—turning the world upside down and inside out, and creating considerable tumult in the process. Callois called the pursuit of vertigo in play *ilinx*, describing it as one of the four major categories of play, in which the player “gratifies the desire to temporarily destroy his bodily equilibrium, escape the tyranny of his ordinary perception, and provoke the abdication of consciousness” [55] (p. 44). Physically, this kind of play results in an increased sense of spatial awareness, vestibular and proprioceptor strength, physical coordination and balance. What is fascinating is that balance is strengthened through the deliberate exploration and experience of imbalance. According to Sutton-Smith, it is the player’s deliberate intent to create and experience this imbalance, to create nonsense out of sense, not necessarily to resolve the opposition, rather in an effort to experience it fully [56]. The deliberateness of children’s intent is echoed in the recent work of Lester and Russell on players’ exploration of risk in play as the deliberate creation of uncertainty [57] (p. 8). 

Locomotor-rotational play involving jumping, leaping, twisting, swinging and running is common in the play of many animal species [29] (p. 340). Spinka *et al*. interpret the function of this kind of play as “training for the unexpected” [42], creating novel behavioural patterns and rehearsing the flexibility of response needed in a rapidly changing environment. While it is also common in young children, it is not generally regarded as beneficial or significant. There are varying levels of adult tolerance for the tumult that is created by dizzy play or chaotic play [58], particularly in indoor environments. Young children’s dizzy play also includes elements of verbal nonsense (which may be rude as well as inappropriate), spontaneous fantasy, and group glee, mentioned previously. These kinds of play are often shut down or only allowed in the outdoor environment. 

Gadamer [37] (p. 106), speaks to the freedom of decision-making that characterizes play for the player and to playing as the exercise of free impulse, a notion that contrasts with emerging evidence in neuroscience about the role of play in refining the executive functions in the prefrontal cortex of the brain, and specifically, with our ability to control impulses [59]. Ironically, it is through the experience of exercising free impulse in play that young children improve impulse control. One of the durable insights from the research on rough and tumble playfighting is that it does not tend to lead to real fighting [44]. It is fascinating that play that looks aggressive actually builds social empathy and emotional self-control, and may prevent the development of aggression [60]. Studies of rough and tumble play in young children reveal that it is an expression of caring and friendship [61]. It is tempting to theorise that play works by providing an experience of the opposite, however this does not explain its generative power. In play, the players explore the dynamic space—the play—between order and disorder, reordering and rebalancing themselves in relationship to the experience and to other players, finding just the right balance in the moment. Sometimes order becomes disorder; sometimes disorder becomes a new order. As Sutton-Smith points out, order and disorder in play are not opposites; they are ambiguities [25]. The power of play is in the moment and the ongoing resolution of imbalance is dynamic, unique to the player(s) and often fleeting and momentary. A crawling infant playing with a ball is motivated by its unpredictability and rewarded by its responsiveness. The infant experiences a sense of agency, power and influence, simultaneously with unpredictability, uncertainty and lack of control of the movement of the ball. The result is fun. 

It is significant that play is behavior in the “simulative mode” [48], separated from its real consequences. Playing creates an intense and immediate emotional reality, one that provides an opportunity for the player(s) to experience and respond to the real feeling of terror for example, without the reality of a predatory threat. This dimension of play is observable in mother-infant play across species, where, as Jerome Bruner observes “the mother seems able to bring the young, so to speak, to the edge of terror” [62] (p. 48). This quality of play makes it safe to take a chance: “When we play, we prod the world—and ourselves—to discover our limits. We willfully put ourselves in precarious situations so that we can experience the emotions that attend success and failure, danger and security. In so doing, we see more clearly the spectrum of our own possibilities” [63] (p. 1). 

The experience of joy and freedom and thrill that many of us associate with our memories of childhood play are the possibilities that draw children into play, possibilities that frequently involve disorderly, disruptive elements of risk, uncertainty and unpredictability—social and emotional, as well as physical. The player is taking a chance that the risk might connect them to the world and others in new ways, and when it does, the player feels powerful. The understanding emerging from neuroscience that “playing is a way of building and shaping the emotion, motivation and reward regions of the brain” [4] (p. 15) resonates. 

There is a body of knowledge coalescing around the social and emotional value of the rough and tumble dimensions of spontaneous free play. Rough and tumble play is common in children, adolescents and across many animal species. While there is now a substantive body of evidence pointing to the benefits of playful aggression in young children [64], it remains one of the most challenging kinds of play to support in group environments. Many do not recognize that rough and tumble playfighting is social play. Gender does appear to play a role; rough and tumble playfighting is more common amongst boys than girls and it is more difficult for women (particularly women who have not experienced rough and tumble play themselves) to distinguish this kind of play from aggression [65]. Research with animals, as well as children, confirms that the signs and behavioural action patterns of rough and tumble playfighting are distinct and recognizable. Maintaining reciprocity is essential and this requires that players exercise self-restraint and engage in continual role reversals and self-handicapping actions. Players actively seek to balance the relationship in order to keep the play going. 

The recent work of Pellis *et al.* [46,49,60,66] on rough and tumble play in rats adds compelling new dimensions to our understanding of this pervasive form of play. Their close and systematic observation of rough and tumble playfighting in juvenile male rats, accompanied by neuroscientific analysis of its impact on the brain, reveals that in rats, the experience of peer to peer play fighting in the juvenile period “can lead to organizational changes in the brain, especially in those areas involved in social behavior” [49] (p. 95). They demonstrate that rats deprived of play fighting in the juvenile period display significant deficits in social competence as adults. They conclude that the significant contribution of rough and tumble play fighting to the normal social development of juvenile rats is the opportunity it provides for rats to “co-ordinate their movements” with a peer. One of the remarkable insights from this research is what it reveals of the subtlety and complexity of the social communication and social cues that characterize rough and tumble play. Recently, when confronted with the ambiguity and subtlety of the social communication in rough and tumble play, young children in our child care lab school agreed that “stop—for real” meant that the play was going too far, while “stop” was considered necessary to the playful exchange and did not indicate that you really wanted to stop playing [67]. This kind of nuanced co-ordination of self with others is, in some sense, the basis of participation in social life. That children might learn to do this through full body contact with others in what may appear to be aggressive behaviour, is a significant insight.

Pellis and Pellis [49] (p. 97) argue that there is now sufficient evidence of continuity between playfighting in human and non-human animals to support the notion that rough and tumble play may promote the development of social competence in children. This finding confirms earlier research with children which established a correlation between social competence and children who frequently engage in rough and tumble play [68]. There is growing interest in looking at the implications of these findings for early childhood care and education [69], and new research is emerging. A recent study of rough and tumble play in Norwegian preschools [70] builds on the notion of affordances in the environment—physical, social, and relational affordances—that invite or inhibit, condone or prohibit rough and tumble play. Freeman and Brown argue that it is time to reconceptualize rough and tumble play in early childhood education, to “ban the banning,” and intentionally welcome consensual rough and tumble play [71] (p. 230).

To summarize, in early childhood, spontaneous free play promotes social and emotional health and a subjective sense of control, agency, well-being and belonging—in the here and now. Burdette *et al*. provide an alternative conceptual framework for describing the immediate benefits of spontaneous free play in health, arguing that spontaneous free play contributes to *attention* (self-control), *affiliation* (friendship), and *affect* (happiness) [19]. The improvisational nature of spontaneous free play builds flexibility and resilience in emotional and social responses, contributing to adaptability in the moment. The simulated, intense and immediate reality of play provides a unique vantage point from which children can explore the social and emotional world, one that invites them to appreciate and experience serendipitous joy, as well as become comfortable with unpredictability and uncertainty. The experience of rewarding, successful and satisfying participation in social play with friends, initiated, directed and sustained by young children themselves, and including the disruptive, rambunctious rowdy dimensions of full body social play contributes to a robust and resilient sense of social connectedness that is essential to long term physical and mental health. 

## 3. Spontaneous Free Play in Early Childhood Care and Education

Spontaneous free play promotes social and emotional health in early childhood. There is an urgent challenge before us in early childhood care and education to clearly identify and then consistently create the conditions that allow spontaneous free play to flourish in early childhood care and education programs. What enhances spontaneous free play? As Kalliala notes:

Supporting children’s play is more active than simply saying you believe that it is important. When children’s play culture is taken seriously, the conditions which make it flourish are carefully created. Children’s play culture does not just happen naturally. Play needs time and space. It needs mental and material stimulation to be offered in abundance.

[72] (p. 139)

Group settings in early childhood may offer young children new opportunities to explore and develop spontaneous free play, however they present significant challenges. The spontaneity of spontaneous free play is critical to its health benefits, as is the control of the play by the players. Is it possible to support spontaneity in free play in a formal early childhood environment, bound as it is by regulations, occupational and accreditation standards, and early learning outcomes? How do early childhood educators plan for spontaneous free play? How do we create the conditions that make it possible for three-year-olds to control and direct their own play? Is a pedagogy of play possible without destroying play [73]? These are critical questions right now in early childhood pedagogy.

Notions of child-centred care and education, the centrality of play and “learning through play” have become deeply embedded in the philosophical foundation and professional practice of early childhood care and education. Based in developmental science, play is understood to be essential to development, and to have universal and naturally occurring benefits, illustrating what play scholars have labelled as the “play ethos” [3,25]. This theoretical foundation is criticized in early childhood pedagogy for its tendency to romanticize all play as innocent, pure, and positive, ignoring or shutting down common play experiences that explore more complex and problematic dimensions of our humanity, like social power, gender roles, and racial diversity [74,75,76,77]. Many early childhood scholars argue that it is time to reflect more critically on the taken-for-granted approaches to play that have evolved in early childhood care and education, defining an intentional pedagogy of play that considers these complex issues and clearly articulates the rationale for a continued pedagogical focus on spontaneous free play, as well as the conditions that support and enhance it in formal early childhood care and education programs [78,79,80]. 

What does it look like when early childhood educators focus intentionally on providing opportunities for children to play on their own terms and for their own purposes? How does it change the role of the educator? What conditions create the possibility of extended episodes of spontaneous free play, controlled and directed by young children? With these questions in mind, I have been following the work of Anne and Brittany, two early childhood educators in a university child care laboratory school, whose collaborative focus over the past several years has been to understand the meaning of the child as a citizen, with a right to play for their own purposes. Each has become deeply involved in reflecting on the meaning of play from the perspective of the young child. Their work draws on new thinking in early childhood pedagogy, connected to the image of the child as a strong, resourceful, capable learner and citizen, and the role of the early childhood educator as a co-learner and co-researcher alongside children and families, in a democratic play and learning community [81]. Following is a preliminary discussion of the possibilities that emerge when educators critically reconsider taken-for-granted practices of provisioning the environment for indoor play, refocussing their participation on co-creating the relational space and time for spontaneous free play alongside children. 

### 3.1. Towards a Pedagogy of “Organized Chaos”

The notion of play as a pedagogy of organized chaos is borrowed from Somerville and Green’s study of outdoor learning in primary schools in socio-economically disadvantaged rural communities in Australia [82]. Building on the ideas of ecological learning and place-based education, as well as other critical pedagogies, the authors conceptualize that deep ecological learning results from a pedagogy of organized chaos, one that calls upon the educator to be “responsibly uncertain,” and “willing to risk the unknown.” The experiences that inform the pedagogy of organized chaos were founded on children’s “immense pleasure with an impromptu, chaotic and random ‘jungle’ adventure,” in the outdoors, where a “sense of imagination and impulsiveness that enables a liberating interaction with the more wild aspects of a school ground landscape” (p. 22) led to rich pedagogical encounters.

Though these ideas are developed in relationship to the exploration of a natural outdoor learning environment, they resonate both with the characteristics of spontaneous free play and its potentially disruptive, chaotic elements, and with the stories of practice that follow. Thinking about play as a pedagogy of organized chaos may open up new possibilities for honoring children’s purposes in play and their unique ways of organizing themselves in play. 

#### 3.1.1. Citizenship and Community are Critical Conditions for Spontaneous Free Play

Spontaneous free play does not just happen; it thrives when children have strong and trusting relationships with one another. Based on her multiyear research on children’s citizenship in early childhood programs [83] and her abiding belief in the value and capacity of children to control their own play, Anne speaks about notions of citizenship and community as critical conditions for spontaneous free play to flourish. The environment for play is much more than a physical reality for children. There is a cultural environment that supports young children’s play, one that requires intentional work on the part of early childhood educators and that takes time to develop in group settings. Anne describes her role in providing rich materials and resources for children’s play, “firing up their imaginations” with stories and new information. The group of children she works with this year have been fascinated by superhero play; after careful thought, she brought in a children’s story book about Achilles to stimulate children’s thinking about superheroes in new ways, beyond their experience with TV and movies. Anne puts a lot of energy into building a repertoire of social strategies with children, which allow them to enter and leave play, and to participate actively. Anne and Brittany emphasize the importance of working with children to create an environment of strong social relationships, where each child can trust that their ideas will be taken seriously by the group. Taking the time to build trusting social relationships in each group of children enhances the capacity of young children to engage deeply in extended and continuing episodes of spontaneous free play that they can control and direct independently. Anne emphasizes that they must be able to trust one another before they can play together. Creating a community that values each child as a citizen requires careful listening on the part of the educators and ongoing attunement to how children are negotiating their friendships and play relationships with one another. It does not mean that all children have to play together all the time. And it is not always smooth. 

Both Anne and Brittany maintain that there is a subculture of play that emerges and develops in each group of children. In contrast to a formal school environment, where children move up the grades as a cohort, Brittany comments that in child care “things linger.” On average children will spend three years in a preschool room, entering as three-year-olds and leaving as five-year-olds. Children learn to play from other children; multiage groupings support spontaneous free play by feeding the peer to peer transmission of play culture. Childcare programs create the possibility of multiage play. Recent experimentation at the lab school involved toddlers joining the preschool group for several hours each day. This reinforced the educators’ understanding of the possibilities of multiage groupings to support spontaneous free play, not only stimulating the toddlers’ sociodramatic play in new ways, but also reminding the preschoolers of the pleasure they experienced in sensory play as toddlers [83] (p. 27). As the sense of place and belonging in community is strengthened, so too are the opportunities for rich and varied spontaneous free play experiences. 

#### 3.1.2. Co-Creating Places for Play

Both Brittany and Anne maintain that the spaces and places for play must be co-created by the children, and further, that the building and transforming of the space is continual, evolving and changing along with the play. Brittany’s pedagogical teacher research over the past year has been focused on understanding how preschool children create a sense of *place* through their play and how this sense of place in turn influences their play. Through critical reflection on ongoing documentation of children’s play, Brittany observed a common story line in all of the children’s play narratives early in the year. Whether the play characters were dinosaurs or meerkats or superheroes or human beings, the stories were always about family relationships, and the action primarily centred on going places—for example, to and from child care, or to and from the grocery store—common experiences in children’s everyday lives that they were playing and replaying together, creating shared meaning. This is another example of Geertz’ notion that play is a “story that the players tell themselves about themselves” [54] (p. 674). 

After reflecting on this observation with a faculty mentor in an effort to identify possibilities for extending this play, an interesting idea emerged, one that sustained a series of connected play experiences over several months. The idea was to build and add a simple wooden door frame to the playroom, thinking that it might support the comings and goings of children’s play narratives. The doorway has been in the playroom for several months now. Initially, the children did not do much with it, but over time, they began to use it intentionally, not just in the literal sense of defining their movement from one place to another, but also as a portal to another world and even to another identity. The doorway was draped with fabric that began to have specific meanings for this group of children. For example, when children drape the doorway with sparkly material, they use it to enter a fantasy world. 

The practice in many early childhood programs has been to organize the environment for spontaneous free play by providing play centres, for example a house play centre, a dress up centre, a block play area, a quiet play area, and a gross motor play area—provisioned with suitable loose parts, play props and other materials. Although this organization of the environment does offer free play opportunities for children, it also constrains the serendipitous aspects of spontaneous free play, which tends to combine multiple forms and narratives simultaneously. Structuring spontaneous free play in these ways communicates specific messages to children about what to play—in the house centre children are expected to engage in dramatic or sociodramatic family role play—and how to play—rough and tumble play is not acceptable in the house centre; it belongs in the gross motor play area. Through critical reflection on how these environments both invite and limit spontaneous free play, Brittany and Anne have begun to explore a different organization of space, one that values and invites continual transformation. Brittany’s immediate observation was that in this kind of environment children begin to use the whole room to develop and sustain their play. It means that children can negotiate the use of space for different and perhaps unusual purposes. For example, recent family play led to the creation of a sleeping area in the entrance to the playroom. Typically, this would be restricted to the house corner.

These are preliminary reflections. Further theoretical insight on co-creating the space for play with young children may lie in Russell’s recent writing on “co-producing” the space for play in adventure playgrounds [84], Sawyer’s study of the “polyphony” of play that unfolds in an early childhood playroom [38], and Marg Sellers’ fascinating Deleuzian interpretation of the multiple and intersecting “lines of flight” that emerge in children’s spontaneous free play [85]. As Anne describes it, children begin to “see possibilities” in one another’s play and they actively seek to connect their play narratives, sometimes in very creative ways. Sometimes these intersections emerge as a result of a social problem, for example, the need to share space or materials with one another. Anne and Brittany gave me numerous example of this kind of intersecting play—one was about a group of children playing out a narrative of being a film crew, who came upon a group of players who were playing at being cooks and invited themselves for lunch, giving flight to a more elaborate play narrative. 

Young children’s spontaneously occurring play sometimes does not look like much to an adult—it is often repetitive and the narrative sequence is not particularly logical—which makes it difficult to know when it is meaningful to participate. Brittany reflects on the times when the multiple intersecting episodes of spontaneous free play taking place simultaneously in the whole room have become chaotic creating “uncertainty” and “unknowing,” for her as an educator. She has found it sometimes difficult to follow the storyline even when she is listening closely. She can see when it makes sense to the children, but it is not always understandable to her. Anne says we have to trust children to play. We have to wait and listen and try not to interrupt. Lester *et al*. recently proposed that we rethink the role of the participant observer in children’s play, becoming instead an “observant participant” [86] (p. 8). The notion of an observant participant makes clear the impossibility of observing without influencing, drawing attention to the idea that young children’s play spaces are always and inevitably co-produced, and to some degree, controlled by adults. The notion of the observant participant also suggests observing carefully before participating more actively, intentionally seeking a deeper understanding of the meaning of the play for the player before interrupting or redirecting it. This shift in role may open up a legitimate space for recognizing and valuing the disruptive dimensions of children’s play in early childhood programs. 

#### 3.1.3. Honoring Children’s Purposes: Keeping the Play Going

Even very young children have a right to play for their own purposes. Spontaneous free play is enhanced when adults actively seek to understand, acknowledge and respect children’s purposes in play and participate intentionally in ways that keep the play going. Brittany speaks to the importance of children being free to choose an identity in play. In order to support spontaneous free play, she has been exploring the possibilities of honoring that identity though the transitions and routines of the day, and not just during free play time. If a child arrives in the morning announcing that he/she is Batman, she honors this identity at the lunch table and when the child wants to wear a cape outdoors. 

Many of the family play narratives in Brittany’s playroom this year revolved around animal families. In particular, one group of children spent several weeks returning frequently to a pretend play scenario where they became kittens, rolling around on the floor with one another, and rubbing up against the legs of the child care educators, looking to be stroked and petted. This play involved boys and girls in repetitive episodes of very gentle, full body rough and tumble floor play, similar to the gamboling play of kittens. The mama cat appropriated a box of Q-Tips from the art table nearby, which spontaneously became the kitty food. She brought bowls from the house play corner to fill with the Q-Tip food, and the kittens pretended to eat the food by picking up the Q-Tips with their teeth and dropping them back in the bowl. An issue arose for Brittany when the children began to pick up Q-Tips from one another’s bowls. Thinking quickly on her feet, she asked if anyone thought the kittens might get sick from sharing their food in this way. A response came immediately and spontaneously from one of the players, who suggested to all the others that the kittens could eat with their feet instead of their mouths. And the play continued, albeit with a new and “quirky twist” [25] on kitten behavior. What mattered to the children was finding a way to keep the play going. Brittany’s recognition of this purpose and children’s ongoing enjoyment of their play is significant. Much of her reflection this year was about situations like this one, where the participation of the adult can easily interrupt the rhythm and flow of the play, breaking the play frame. In this case she participated as a player might, contributing her thought within the metacommunicative structure of spontaneous free play, where children slip in and out of character roles to shape and direct the ongoing action. 

## 4. Conclusions

The UN Convention on the Rights of the Child calls upon us, individually and collectively, to honor and uphold the image of the child as a participating and contributing citizen, with a right to play, a right “that is most distinctly children’s” and that “defines, almost, the right to be a child” [4] (p. 8). There is good evidence now to support the notion that spontaneous free play contributes positively to mental as well as physical health in early childhood. These benefits are linked to spontaneous free play that is controlled and directed by children themselves; to critical new understanding of the role of rough and tumble play in the development of healthy social relationships; to the capacity of sustained involvement in socio dramatic play to enhance emotional and social self-control; and to the significance of the disruptive dimensions of spontaneous free play in enhancing children’s flexibility and adaptability in response to change and unpredictability. This kind of play is often shut down by adults who experience it as noisy, messy, silly, chaotic, risky, uncivilized, dangerous and annoying.

Creating and sustaining the conditions for spontaneous free play in the increasingly formalized environments in which early experience unfolds presents significant challenges for early childhood educators. It is essential that we honor children’s purposes in play, and reflect critically on taken-for-granted assumptions and practices in provisioning indoor as well as outdoor play environments for young children in early childhood care and education. Increased time in peer groups at a young age may also create new possibilities for spontaneous free play in early childhood. Ensuring young children’s right to play will take intentional effort on the part of early childhood educators and families, with the support of and policy makers and allied professionals. 
